# LIVER TRANSPLANT FOR METASTATIC NEUROENDOCRINE TUMORS: A SINGLE-CENTER REPORT

**DOI:** 10.1590/0102-672020230032e1750

**Published:** 2023-07-17

**Authors:** Raquel Lima Sampaio, Gustavo Rego Coelho, Ana Rosa Pinto Quidute, Duilio Reis da Rocha, Carlos Eduardo Lopes Soares, José Huygens Parente Garcia

**Affiliations:** 1 Hospital Universitário Walter Cantídio, Digestive Surgical Unit – Fortaleza (CE), Brazil; 2 Hospital Universitário Walter Cantídio, Liver Transplant Unit – Fortaleza (CE), Brazil; 3 Hospital São Carlos, Liver Transplant Unit – Fortaleza (CE), Brazil; 4 Hospital Universitário Walter Cantídio, Endocrinology Service – Fortaleza (CE), Brazil; 5 Hospital Universitário Walter Cantídio, Oncology Service – Fortaleza (CE), Brazil.

**Keywords:** Carcinoma, Neuroendocrine tumors, Liver transplantation, Neoplasm metastasis, Carcinoma, Tumores neuroendócrinos, Transplante de fígado, Metástases neoplásicas

## Abstract

**BACKGROUND:**

Neuroendocrine tumors are rare neoplasms of uncertain biological behavior. The liver is one of the most common sites of metastases, occurring in 50% of patients with metastatic disease.

**AIMS:**

To analyze a clinical series in liver transplant of patients with neuroendocrine tumors metastases.

**METHODS:**

A retrospective descriptive study, based on the review of medical records of patients undergoing liver transplants due to neuroendocrine tumor metastases in a single center in northeast Brazil, over a period of 20 years (January 2001 to December 2021).

**RESULTS:**

During the analyzed period, 2,000 liver transplants were performed, of which 11 were indicated for liver metastases caused by neuroendocrine tumors. The mean age at diagnosis was 45.09±14.36 years (26–66 years) and 72.7% of cases were females. The most common primary tumor site was in the gastrointestinal tract in 64% of cases. Even after detailed investigation, three patients had no primary tumor site identified (27%). Overall survival after transplantation at one month was 90%, at one year was 70%, and five year, 45.4%. Disease-free survival rate was 72.7% at one year and 36.3% at five years.

**CONCLUSIONS:**

Liver transplantation is a treatment modality with good overall survival and disease-free survival results in selected patients with unresectable liver metastases from neuroendocrine tumors. However, a rigorous selection of patients is necessary to obtain better results and the ideal time for transplant indication is still a controversial topic in the literature.

## RESUMO

**RACIONAL:**

Os tumores neuroendócrinos são neoplasias raras de comportamento biológico incerto. O fígado é um local comum de metástase, ocorrendo em 50% dos pacientes com doença metastática.

**OBJETIVOS:**

Analisar casuística de transplante hepático por metástases de tumores neuroendócrinos.

**MÉTODOS:**

Estudo descritivo retrospectivo com revisão de prontuários de pacientes submetidos a transplante hepático por metástases de tumores neuroendócrinos em um único centro no Nordeste do Brasil durante 20 anos (janeiro de 2001 a dezembro de 2021).

**RESULTADOS:**

Durante o período analisado, foram realizados 2.000 transplantes hepático, sendo 11 indicados por metástases hepáticas de tumores neuroendócrinos. A média de idade ao diagnóstico foi de 45,09±14,36 anos (26–66 anos) e 72,7% dos casos eram do sexo feminino. O local do tumor primário mais comum foi o trato gastrointestinal (64% dos casos). Após detalhada investigação, três pacientes não tiveram o local do tumor primário identificado (27%). A sobrevida global um mês e após um ano do transplante foi de 90 e 70%, respectivamente. A sobrevida após 5 anos foi de 45,4%. A taxa de sobrevida livre de doença foi de 72,7% no primeiro ano e 36,3% em cinco anos.

**CONCLUSÕES:**

O transplante hepático é uma modalidade de tratamento com bons resultados de sobrevida global e sobrevida livre de doença, em pacientes selecionados com metástases hepáticas irressecáveis de tumores neuroendócrinos. No entanto, a seleção rigorosa dos pacientes é necessária para obter melhores resultados e o momento ideal para a indicação do transplante ainda é um tema controverso na literatura.

## INTRODUCTION

Neuroendocrine tumors (NET) are rare neoplasms of uncertain biological behavior. Its clinical presentation varies in local and indolent form or, in some cases, with metastatic dissemination and clinical syndromes related to hypersecretion of hormones or bioactive amines. Metastases to the liver are common, occurring in 50% of patients with metastatic disease^5,9^.

The role of liver transplantation (LT) in patients with NET metastases, especially regarding the ideal time to be performed, is still controversial in the literature^5,8^.

The objective of the present study is to analyze the experience in LT of patients with NET metastases in a large center in the northeast region of Brazil.

## METHODS

This is a retrospective descriptive study, based on medical records of patients undergoing LT due to neuroendocrine tumor metastases in a single center over 20 years (from January 2001 to December 2021). The study was approved by the Institution's Ethics Committee (n° 5.187.244).

## RESULTS

During the analyzed period, 2,000 LT were performed by the team, of which 11 were indicated due to NET liver metastases. The mean age at diagnosis was 45.09±14.36 years (26–66 years) and 72.7% of cases were females. On average, the patient's BMI was 23.9±14.36 (18.03–31.25). Blood type O was the most frequent (45%), followed by types A and B (27% each).

The most common primary tumor site was in the gastrointestinal tract in 64% of cases. Even after detailed investigation, three patients had no primary tumor site identified (27%). One of the first cases in the service was a patient with a primary neuroendocrine tumor of the cervix. Except for the patients with unknown primary tumors and one with pancreatic NET, all of them underwent a previous surgical approach with resection of the primary tumor site. The Ki-67 levels of the primary tumors ranged from 1 to 5%. All 11 patients had metastases in the liver and five (45%) developed carcinoid syndrome.

Before LT, somatostatin analogs were administered in 72% of patients. A patient with cervical NET underwent chemotherapy, followed by partial hepatectomy. All patients had multiple foci of metastatic involvement restricted to the liver.

The interval between the surgical approach to the primary tumor site and LT ranged from one year and five months to 13 years. Only one case had the primary tumor site approached during the transplant, and a total pancreatectomy was performed.

The mean Model for End-Stage Liver Disease (MELD) score calculated was 7.4. The mean cold ischemia time was 285±110 minutes (154–446 minutes). The mean warm ischemia was 31±8 minutes (20–44 minutes). Only three patients required a transfusion of blood concentrates in the transplant. No patient had intraoperative complications.

Overall survival at one month and at one year after transplantation was 90% and 70%, respectively. Two patients had tumor recurrence, one presenting with peritoneal metastasis and the other with brain metastasis. Survival was significantly lower in the primary tumors outside the gastrointestinal tract. One patient died after detecting a brain lesion during follow-up in the first year after transplantation. Unfortunately, it was impossible to perform a brain lesion biopsy to distinguish between a metastatic lesion and a primary lesion of the central nervous system. Two patients died of sepsis.

The overall survival rate was 72.7% in the first year and 45.4% in five years. Disease-free survival rate was also 72.7% in the first year and 36.3% in five years.

## DISCUSSION

A significant increase in the incidence of NETs has been observed in the last four decades, with an annual incidence of approximately 3.6–3.9 cases per 100,000 inhabitants, representing about 5% of gastroenteropancreatic NETs^2^. The increased use of imaging tests has been an important cause of this rise in the incidence rate, detecting asymptomatic lesions earlier^1^.

Carcinoid syndrome is frequent in patients with liver metastases from NET, from the primary tumor site to the midgut (20–30%), characterized by tachycardia, skin flushing, bronchospasm, diarrhea, hypotension, fibrotic complications such as mesenteric and retroperitoneal fibrosis, and cardiac involvement. The syndrome's pathophysiology is associated with tumoral hypersecretion of peptides, vasoactive amines, and prostaglandins not inactivated by the liver due to metastatic involvement^2^.

In cases of liver metastases, surgery is indicated when it is possible to perform complete resection in the absence of extrahepatic metastases, bilobar involvement, or impairment of liver function ^1,3,4,8^. Although in large series, symptoms control occurred with resection of metastases in 96% of cases and recurrence of symptoms happened in 59% within five years. The tumor recurrence rate in patients with unilobar involvement or single metastases was approximately 80% and 95%; the overall survival was 61% and 35% at five and ten years, respectively^1,4^. Thus, as an R0 resection is rarely curative, LT seems a good alternative in selected cases^1,8^.

The role of LT in patients with NET metastases is still controversial, mainly because its indication has improved in recent decades, with a limited series of prospective studies. Transplantation by NET metastases corresponds to approximately 0.2–0.3% of transplant indications in the US and Europe^1,3^.

The two largest published case series are an American study with 184 transplants and a European with 213. Overall survival rates at one, three, and five years varied between the two studies at 79–81%, 61–65%, and 49–52%, respectively^1,4^. In the European study, the disease-free survival rates at one, three, and five years were 65, 40, and 30%, respectively, showing a considerable recurrence rate but lower when compared to isolated liver resections^3^.

A recent study of 88 transplant-eligible patients showed better overall survival in the transplantation group than in the group that did not undergo transplantation (five-year overall survival of 97.2 vs. 88.8% and 10-year survival of 50.9 vs. 22.4%)^1,6^. In addition, despite possible study selection bias, it was possible to observe that the benefit of transplantation increased with time, with an approximate survival gain of 38.4 months after ten years^1,6^.

Due to the insufficiency of donors in general, the indication for transplantation must consider the risks and benefits of the procedure, with an appropriate selection of patients^3,7^. Based on multicentric cumulative experiences, Mazzaferro et al. introduced the Milan criteria for NET metastases, a vital guideline used to select candidates for transplantation^4,6^. Based on these criteria, the Milan group reported five-year overall and disease-free survival rates of 97 and 89%, respectively^1,4,6^.

In a review of the clinical criteria associated with LT in patients with recent NET metastases, Kim et al. found the main prognostic factors are Ki-67 and hepatomegaly^3^.

Although a percentage of less than 50% of liver involvement is one of the criteria for transplant indication, Olausson et al. showed promising results in a series of 15 patients undergoing transplantation (10 liver and five multi-visceral), all with liver parenchymal involvement above 50%, with a five-year overall survival of 90%^6^.

The ideal time to suggest transplantation remains controversial. Some authors emphasize that transplantation should be reserved for cases in which local therapies (surgical resection, radiofrequency ablation) are not indicated, and for a minimum period of six months of disease stability, which is advocated by most other authors, for a better understanding of the tumor's biological behavior^3,6^.

## CONCLUSIONS

LT is a treatment modality with favorable overall survival and disease-free survival results in selected patients with unresectable liver metastases from NET. However, a rigorous selection of patients is necessary to obtain better results. Besides, the ideal time for transplant indication is still controversial in the literature.
